# Editorial: Mental health in correctional and criminal justice systems (CCJS): exploring how diagnosis, treatment and cultural differences impact pathway through the CCJS

**DOI:** 10.3389/fpsyt.2023.1293060

**Published:** 2023-10-31

**Authors:** Nigel McKenzie, Andrew Forrester

**Affiliations:** ^1^Division of Psychiatry, University College London, London, United Kingdom; ^2^Department of Psychological Medicine and Clinical Neurosciences, School of Medicine, Cardiff University, Cardiff, United Kingdom

**Keywords:** correctional and criminal justice systems, mental health, met and unmet treatment needs, suicide among prison inmates, continuity of care on release from prison, prison inmate

A number of previous reviews of the literature have shown the prevalence of mental disorders in prisons is considerably higher than in the general population (1–3). This includes data from low and medium income countries as well as higher income countries. One study (4) which assessed data from 13 low and middle-income countries found the prevalence of non-affective psychosis was on average 16 times higher than the general population, major depression and illicit drug use disorder were both six times higher, and the prevalence of alcohol use disorders was two times higher. Findings from high-income countries are similar.

In regard to the treatment of prisoners with mental disorders, a policy document from the WHO “Good governance for prison health in the twenty first century” (5) recommends that the health care of prisoners should be supervised by government in the same way as the general population. This is also in keeping with the United Nations Standard Minimum Rules for the Treatment of Prisoners—the Mandela Rules. Rule 24 states that “the provision of health care for prisoners is a state responsibility” and that “prisoners should enjoy the same standards of health care that are available in the community, and should have access to necessary health-care services free of charge without discrimination on the grounds of their legal status” (6). Few studies have examined the mental health care received by prisoners. Another issue is deaths which occur while in prison custody or shortly afterwards. This is an ongoing issue that needs to be resolved, with more research, and better understanding of interventions required to improve the situation (7–9).

Where studies have been undertaken they have indicated the needs for treatment of prisoners for mental health disorders have often not been identified and treatment needs have not been met (10, 11). Few studies have examined what factors impact the pathway through the criminal justice system in terms of treatment received and whether needs were met, although some have suggested poorer outcomes for people from ethnic minority groups (12). Another largely unexplored area is the extent to which the treatment needs of prisoners discharged from prison are handed over to appropriate mental health services in the community (13).

Simpson et al. conducted a systematic review of publications investigating mental health services in the correctional framework. In regard to those passing through the correctional system, they found no evidence for standardized assessment approaches, some evidence to support specific psychosocial interventions and relatively weak evidence to support reintegration methods back into the community.

Other contributors to our topic have added to what is known. Linked to the theme of the effectiveness of interventions for those passing through the criminal justice system, Yesuf et al. looked at the prevalence of mental illness among inmates in north-western Ethiopia. They found around 75% of inmates had some form of mental disorder with symptoms including feeling unhappy and finding it difficult to play an important role in life. However, those that took part in rehabilitation activities had an improved outcome. The authors concluded that this was an area for further research and development that could lead to better outcomes for those with symptoms of mental disorder.

Stawinska-Witoszynska et al. found a high level of generalized anxiety disorders (GAD) in the population of inmates detained in one of the largest penitentiary units in north-eastern Poland. They found a three times higher prevalence of GAD among inmates detained in a closed type prison compared with those in an open prison. They made the case for increased availability of psychological therapies for prisoners.

Another theme of this topic is whether care is sufficiently integrated between the criminal justice system and community based services. McIntosh et al. conducted a mental health needs assessment for Scotland's prison population. They concluded that existing provision to support the mental health needs of people in prison in Scotland was inadequate. This was partly due to a lack of integrated care between the justice, health and social work systems resulting in prisoners not receiving the support they needed both during and following imprisonment.

In line with another topic theme some research has been undertaken on treatment options for different mental health conditions seen in prisons in various countries. Sekiguchi et al. looked at treatment options for persistent methamphetamine associated psychosis and how this should be distinguished from schizophrenia spectrum disorder. They found benefits from using a lower dose antipsychotic to treat methamphetamine associated psychosis. Naidoo et al. in a study based on a women's correctional center in South Africa emphasized the need to educate, support and manage those infected with HIV which has a high prevalence in South Africa. In Canadian based studies, Moghimi, Knyahnytska, Zhu et al. explored the mental health needs of correctional workers and found, Moghimi, Knyahnytska, Omrani et al. digital mental health care interventions could help meet these needs. Shafti, Steeg et al. and Shafti, Taylor et al. explored the relationship between aggressive behaviors and self-harm and implications for managing such behavior in correctional settings.

In terms of other themes of this Research Topic such as whether an individual's needs for treatment and care are successfully handed over to community-based services and the impact of the quality of care on the likelihood of recidivism more research is needed. As noted by Skipworth et al. a study in New Zealand indicated that there has been an increasingly poor outcome in terms of imprisonment following discharge from a mental health unit over a ten-year period and concluded that models of community based mental health care may be increasingly reliant on the criminal justice system to manage aggressive and dangerous behavior among those with mental illness. There have been reports of similar trends in other countries including the UK.

## Author contributions

NM: Writing—original draft. AF: Writing—review and editing.
